# Patient-oriented research competencies in health (PORCH) for researchers, patients, healthcare providers, and decision-makers: results of a scoping review

**DOI:** 10.1186/s40900-020-0180-0

**Published:** 2020-02-10

**Authors:** Noreen Frisch, Pat Atherton, Mary M. Doyle-Waters, Martha L. P. MacLeod, Anastasia Mallidou, Vanessa Sheane, John Ward, Jinelle Woodley

**Affiliations:** 1 School of Nursing, University of Victoria, 3800 Finnerty Rd, Victoria, BC V8P5C2 Canada; 2 BC SUPPORT Unit, Suite 420 1367 W Broadway, Vancouver, BC Canada; 3 Centre for Clinical Epidemiology and Evaluation, 708 - 828 W. 10Th Avenue, Vancouver, BC Canada; 4 University of Northern British Columbia, 3333 University Way, Prince George, BC Canada; 5 Island Health Authority, 1952 Bay St, Victoria, BC Canada

**Keywords:** Patient-oriented research, Patient and public involvement, Patient engagement in research, Competencies, Scoping review

## Abstract

**Plain English summary:**

**Background**

The Canadian Institutes of Health Research funded a program, “patient-oriented research” (POR), to change the way health research is done. POR involves patients and their families/caregivers as equal partners on research teams with researchers, healthcare providers and decision-makers. The authors of this paper work through a unit in British Columbia, Canada that functions to help research teams learn how to do patient-oriented research. We felt that we could not train people if we didn’t first understand what others had learned about what competencies (knowledge, skills and attitudes) were helpful for members of these research teams.

**Method**

We used a method called a scoping review to search literature on patient-involved research. Our search included papers in academic journals as well as information on websites, training manuals, conference proceedings, governmental documents and statements from health organizations.

**Findings**

Writers reported the usefulness of many competencies for researchers and patients, with fewer competencies for healthcare providers or decision-makers. The main competencies for researchers had to do with *participation*, *communication* and *conflict management;* for patients they had to do with *research knowledge and skills*, *cultural competence* and *participation*. It was helpful that all team members want to work as part of a group for the public good.

**Conclusions**

We worked with an advisory group of people representing patients and their families/caregivers, researchers, healthcare providers and decision-makers to review our findings. We concluded that our competency statements are helpful for people to determine what they need to know or learn as they join research teams.

**Abstract:**

**Background**

The Canadian Institutes of Health Research (CIHR) launched an initiative called the Strategy for Patient-Oriented Research (SPOR) encouraging patient-oriented research (POR) that engages patients as equal partners in research teams alongside researchers, healthcare providers and health system decision-makers. Other countries have launched similar initiatives (POR-related work) yet there has never been full review of the competencies needed by individuals engaging in this work.

**Purpose and methods**

Our purpose was to summarize existing knowledge on POR and POR-related competencies by conducting a scoping review of peer-reviewed and grey literature. Our objectives were to systematically explore literature, articulate competencies necessary for research team members, identify research gaps and provide recommendations for further research. Using standard health databases and search methods, a total of 2036 sources was retrieved. Data were extracted from 35 peer-reviewed papers and 38 grey literature sources. We used an iterative process to reach consensus on competency statements.

**Findings and conclusions**

The main competencies for researchers were in categories of *participation*, *communication and teamwork* and *conflict/tension management;* for patients the main competencies were in *research knowledge and skills*, *cultural competence/context* and *participation*. While fewer competencies were documented for the other stakeholder groups, the need for *understanding patient involvement in research* and *knowledge of the needs that research partners have* are noted as competencies for healthcare providers and decision-makers. Attitudes demonstrating inclination to conduct the work were noted for all. The competencies can be used to consider learning needs of research team members and for team members wishing to assess their own readiness to serve on a POR or POR-related research team. Incidentally, we noted the lack of a common vocabulary used to describe patient-involved research, a situation making research and literature review/retrieval quite challenging. Recommendations for future research and for achieving consistency in language are addressed.

## Introduction

Patient-oriented research (POR) is the Canadian initiative led by the Canadian Institutes of Health Research (CIHR) to engage patients as research partners in all areas of health research, with patients defined as individuals with personal experience of a health issue as well as their informal caregivers, including family and friends (1). The POR approach focuses on patient-identified research priorities and incorporates into multidisciplinary research teams: (a) patients, (b) researchers, (c) healthcare providers, and (d) health system decision-makers; these make up the four stakeholder groups that define POR as a unique approach to health research, similar to but differing from participatory action research, community based research, and other inclusive approaches, such as professionals and community partners working together to co-produce health research. In POR, patients and community partners can be engaged at varying levels such as those articulated by the International Association for Public Participation (IAP2) http://iap2canada.ca, and they may be involved as partners in priority setting, conducting research, and bringing research findings into clinical practice and health policy. POR and related research is important because partnerships that include individuals in each of the four stakeholder groups working together provides differing perspectives that can lead to establishment of sound and relevant research priorities, discussions of appropriate research methods, interpretation of data from diverse perspectives, and a realistic sense of when and how to move research findings into practice. The goal of POR and related research is achieved when research findings improve the health of the population studied.

In order to build capacity to conduct POR in the province of British Columbia (BC) Canada, a SUPPORT Unit (Support for People and Patient-Oriented Research and Trials, or “Unit” hereafter) was established in 2016. This Unit is a multi-partner organization aiming to “support, streamline and increase Patient-Oriented Research within BC.” http://bcsupportunit.ca/about/. Working as part of the Unit’s training and capacity development effort, we were faced with uncertainties about how to plan, implement and evaluate training without an understanding of the competencies required for POR. We believed that an understanding of these competencies is the essential first step in development of any educational program or mentoring activities. We knew that there are successful and established international approaches to engage patients in health research (such as INVOLVE of the United Kingdom’s (UK) National Health Service and the Patient-Centered Outcomes Research Institute (PCORI) of the United States (US)) however, we found no readily available statement of competencies for patient-involved research. We found references to ‘training’ or using ‘trained patient partners’ and some references to researcher knowledge in use of a patient involved approach, but these were without a description of what content was delivered in training, what learning outcomes were sought, or what skills were being developed. Thus, we embarked on our project to complete a systematic search of the literature to document what others, in Canada and beyond, have written about competencies needed to engage in this work. In planning our search approach to the literature, we became aware of the fact that there is no common language used to refer to patient-involved research. Terms such as the Canadian POR overlap in meaning with terms used elsewhere, such as ‘patient involved research’, ‘patient engaged research’, or ‘patient participatory research’, yet these terms may not be synonymous, or their meaning may be different depending on the geographical location and research culture. We designed our search to include not only POR but all patient-involved research in order to obtain results that encompassed all work in this field. For purposes of this paper, we use the phrase ‘patient-involved research’ as an overarching term to refer to all research in which patients, family members and/or community partners work with a research team to study a health-related issue. We believed that in doing so, we would be able to retrieve relevant literature and gain knowledge of the competencies individuals need to engage in this work.

We adopted an educational approach to competencies that defines a competency to include “combining and mobilizing attitudes, knowledge, skills, and external resources and then applying them appropriately to specific types of situations” (2). Using this competency framework, we wanted to know what knowledge is needed, what skills should be performed and, perhaps most importantly, what attitudes should be held for members of each of our stakeholder groups participating on a POR or POR-related research team.

We conducted a scoping review of the literature to answer our questions. The purpose of a scoping review is to examine literature as widely as possible and to report clearly what the literature on the topic states. A scoping review does not interpret, prioritize, or filter the information presented in the literature in any way. Our author team was comprised of a group of individuals with background and expertise in conducting such reviews and included an experienced health librarian who could guide the search process. Our methods section and our scoping protocol detail our process in finding, retrieving, and reporting literature. Our belief was that patient-partners as well as members of each of the other stakeholder groups could best contribute by assisting us to interpret and evaluate the competencies reported in the literature. Therefore, in the second part of our work where we evaluated and reflected on our data, we asked members of an Advisory Group to examine what we found, tell us if they believed the competencies reported were important, resonated with their experiences, and ‘fit’ with their views of what members of patient-involved research teams needed. Additionally, we asked if any of the advisors thought a critical competency was missing from our list. The Advisory Group was comprised of nine individuals, each having participated in patient-involved research and included two representatives from each of the four stakeholder groups and one individual with extensive background in patient engagement in research and in teaching/mentoring research teams to take up this work.

This paper is organized the following way: 1) we present our methods in conducting the literature search; 2) we report the competencies found in the relevant literature for each stakeholder group, and 3) we present a discussion on the relevance, usefulness, challenges or possible limitations of the competencies reported, based on our reflections, guided by input from our Advisory Group.

## Methods

We used the scoping review approach (3–5) because this method permitted us to search for a wide range of published work, not limiting us to peer-reviewed publications only. Our sources included research papers, descriptive studies, reports of educational and professional development programs, and commentaries published in peer-reviewed journals, and written vision statements, training materials, and reports from institutions undertaking patient-involved research work in the grey literature. The full protocol for this review has been published elsewhere (6); we provide a summary here of the five stages of our review.

### Formulating the research question

Our research question was: *What are the core competencies needed by researchers, patients, healthcare providers and health system decision makers undertaking POR or POR-related (or patient-involved) research? Our objectives were to systematically explore peer-reviewed and grey literature on the competencies for the four stakeholder groups, summarize and articulate the competencies found, provide recommendations for their use, and identify research gaps to suggest areas for further research.*

### *S*creening and identifying the literature

In consultation with our team’s librarian, an iterative search strategy was developed and refined through the search process. Searches were carried out in three stages.

#### Stage 1

Preliminary searches of peer-reviewed literature focused on identifying appropriate key words and terms for our topic. After 11 searches of the Embase, MEDLINE, and Web of Science databases, Google Scholar and key authors writing on the topic resulting in 110 papers, we identified eight to read full-text as these appeared to address research competencies. However, in these 11 searches we found no clear pattern of terms used to present the topic. For example, using a search phrase ‘patient engagement in research’ or ‘patient-engaged research’ led to hundreds of papers on patient engagement in clinical decision-making. Further, in this initial search we found there were no journals that were regularly referring to competencies or specific training activities or learning outcomes in this field that could be used to guide hand searching of specific journals. Thus, our team librarian recommended that we limit our searching to review articles (e.g., systematic reviews, scoping reviews, realist reviews). This recommendation was based on the observation that seven of eight papers that were selected for full-text review in our Stage 1 search were review papers. We believed that capture of review articles would provide the best means to target relevant papers and would allow for review of reference lists of those papers to obtain additional sources. We agreed to search papers from any journal issues devoted to this topic, should any be found, and to follow the writings of key authors on the topic as they emerged in our search. Thus, we amended our protocol to carry out Stage 2 of the search.

#### Stage 2

In this stage we searched review articles through: MEDLINE, Embase, and CINAHL, the reference lists of all identified review articles, one journal issue focusing on this topic, and a reference list supplied to us by the authors of a recently published study (7). We found and included five additional review articles.

#### Stage 3

We searched grey literature through search engines and by reviewing organization websites, identifying 131 sources. Two team members independently reviewed these sources, assessing each for inclusion, and consulted with each other to reach agreement.

### Selecting relevant studies and publications

Publications and grey literature sources were reviewed for inclusion using predetermined criteria:

Published papers from 1/1990–1/2018 in health literature or current grey literature sources that addressed:

- Population: either researchers, patients, healthcare providers, or health system decision makers,
- Concepts: attitudes, knowledge or skills that promote efficacious patient-oriented research,
- Context: patient-oriented research, and;
- Language: English or French.

Team dyads reviewed each article or source to determine whether inclusion criteria were met. Any disagreements about inclusion were resolved through discussion between the two reviewers or, when necessary, with a third reviewer. For peer-reviewed literature, our inter-rater agreement was 88.8% for the initial set of reviews. Four dyads completed reviews and one of the four had a moderate level of agreement (53%), while the others were more consistent (87.8–100%). By the second stage of reviews, agreement between dyad members reached 97% agreement at the title/abstract level. Full agreement was reached for full-text review and data extraction.

### Extracting the data

Data were extracted upon full-text review if the source described competencies for any of the four stakeholder groups. Competencies and contextual data were recorded directly from the paper onto a shared Google Drive spreadsheet. Sources were categorized into peer-reviewed papers or grey literature. For peer-reviewed papers, contextual data included study design, theoretical framework, participant demographics, and study findings. For grey literature, contextual data included type of source, URL, and information about the organization. In both cases, extracted competencies were categorized into ‘knowledge,’ ‘skills,’ and ‘attitudes.’

### Collating, summarizing, and reporting the results

Data from our spreadsheet were summarized in narrative form according to traditional integrative review processes, where reviewers identify significant components of the topic and provide narrative statements to report relevant concepts (8–10). We used content analysis to review statements and group concepts into themes (11).

## Data analysis and findings

### Search results

**Fig. 1:**
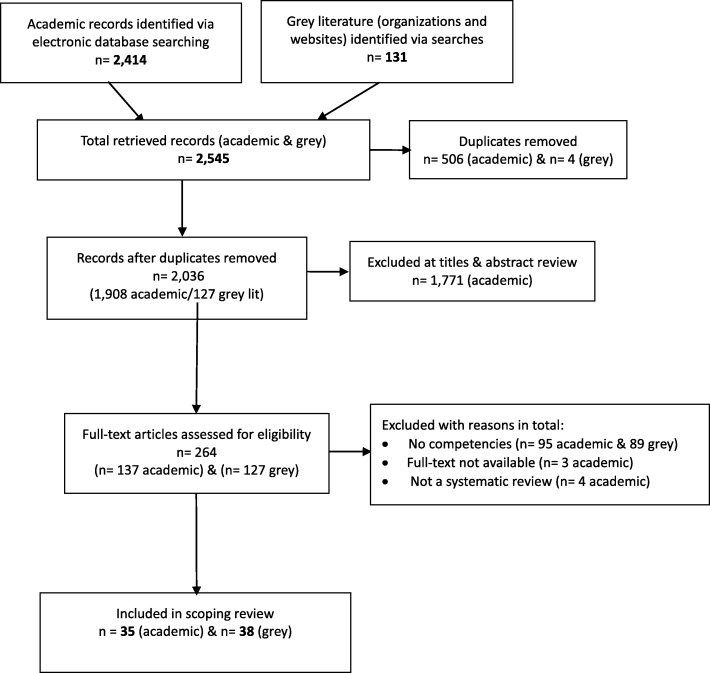
Summary and Flow Diagram of the Search Process Used in the Scoping Review

The combined search processes for peer-reviewed literature resulted in 2414 references from various sources, of which 506 were duplicates, yielding 1908 titles for review. After abstract review, 137 papers were selected for full text review and data were extracted from 35 of these. For grey literature, information from 131 sources was evaluated for full-text review and data were extracted from 38. Figure 1 presents the results of searches.

### Description of included sources

Of the 35 peer-reviewed papers, 16 were from the UK, 11 from the US, five from the Netherlands and three from Canada. Of the 38 grey literature sources, 25 were from the UK, seven from Canada, and others from Australia, the US and international organizations. This distribution of countries likely reflects the extent of interest in the topic over time and recognizes the UK as an early adopter and international leader in patient involvement in research. The peer-reviewed papers included research as well as reflections on experiences of patient involvement, though there were only five papers presenting research findings specific to competencies. The grey literature included governmental material, training materials, information from organizations’ websites and conference proceedings. The terms used in the literature were varied and included ‘patient engaged research’, ‘patient-involvement in research’, ‘patient-oriented research’, as well as ‘community partnered research’, and ‘participatory action research’. We have included the findings of all such papers as these are POR-related approaches.

### Data analysis

When analyzing the data extraction tables, we used conventional content analysis (11) to summarize the data, describe information, and identify emergent themes. The purpose of a content analysis is to describe the ideas, themes, and meaning in text data. For our project, the published papers provided the text data and the content analysis method was used to articulate the themes described or implied in the papers read. A conventional content analysis focuses on a description of what is in the text which fit well with purpose of our work. We reviewed the reported competencies recorded on our spreadsheet and identified their meaning; those with similar meaning were grouped, and themes identified. Team members reviewed and grouped competencies together during a meeting by projecting data tables onto a shared screen and reading each competency aloud. Key words were suggested to articulate the meaning of each competency, allowing for an initial grouping. Consensus was achieved through an iterative process and groupings were refined into themes before being matched to their respective attribute. The reader should note that our complete data and supplemental files are publicaly available located at the University of Victoria repository that can be accessed at: https://dspace.library.uvic.ca//handle/1828/10917

Below, we present the themed competencies of knowledge and skills for each stakeholder group, followed by the attitude competencies, which were not grouped into themes. Additionally, our findings also yielded a set of attributes, related to – but distinct from – attitudes. We report these in the ‘attitudes’ section. We supply the references from the both the academic and grey literature for every competency listed. The competencies under each theme are organized so that the competency that was noted most frequently in the literature is reported first. The reader is cautioned to understand that the competencies are findings from our scoping review and reflect published statements from persons writing on the topic. These do not necessarily represent a global consensus of the knowledge, skills or attitudes/attributes needed for POR or patient-involved research. In keeping with our purpose of presenting findings from a complete scoping review, a competency that may have been noted only once is still reported as part of the data we reviewed. The interpretation and sense of appropriateness or usefulness of these competencies is addressed in our Discussion section.

#### Researcher stakeholder group

*Seven themes were identified for knowledge competencies and six themes for skills.*

##### Knowledge competencies

***Research Methods and Principles****:* Researchers know the research method being used at an expert level. Additional knowledge competencies are those for the following: define the research process (12–14)^;^ state research goals (15); understand logic models (16); analyze data (17); and, have familiarity with research approaches congruent with patient and community engagement (15, 18, 19). Additionally, a report concluded that researchers know strategies for successful participant recruitment and informed consent processes (20). Researchers are expected to understand participatory research is an approach to research and not a method in and of itself (19).

***Participatory Approach****:* Researchers understand participatory approaches and methods (15, 21–29); how to implement them (21); and, are knowledgeable about how to engage patients and public partners in research (22). Knowledge related to the involved community was reported as comprehension of community knowledge (30); understanding of community engagement (30); community involvement (25); the value of community involvement (31, 32); and, knowing how to work with patients and community members (15, 29). Knowledge to work with community partners included understanding the need to balance patients’ right to participate while ensuring patients were not overburdened or exploited (29); understanding the necessity of balancing patients’ perspectives with those of others on the research team (33); and, knowing how to match people to the needs of the research approach (34).

***Understanding Evidence and Results****:* Researchers understand that patients require the presentation of research results in a manner that is readily understood and meaningful (35).

***Cultural Context and Competence****:* Researchers need to know the basics of cultural competence (21); understand the principles of conducting work (such as obtaining informed consent) from within the cultural perspectives of the participants (36); and, to understand the community of interest and its stakeholders (37). Researchers identify their own personal experiences and social contexts related to the study context (38). As researchers embrace new ways of doing research, researchers understand ways in which organizations change and transform to accept differing practices (16).

***Logistics:*** Researchers understand and appreciate the additional time required for collaboration in the research plan (12, 39) and the budgetary implications for involving and compensating patients (39).

***Priority and Agenda Setting****:* Researchers know how funding priorities are set (40); how to communicate these priorities (14, 35); how to determine best new research ideas and future directions (26); and, how to set research priorities that are important to patients (41).

***Understanding POR or POR-related research:****R*esearchers know the relevance and benefits of patient involvement, and understand ways in which patient involvement leads to research more likely to be put into practice (31, 35, 42, 43). Researchers understand the difference between patient centeredness and patient engagement (44), and have knowledge about POR, its guiding principles and its benefits (45).

##### Skills competencies

***Participatory Skills:*** Researchers have the ability to create a safe and respectful environment for all team members (46); attract, recruit and enrol patient and public members in the research process (21, 47, 48); ensure patients have sufficient training to participate (29); see that all information given to the patients is comprehensive and understandable (46); and, involve patients in all aspects of the study design (49). As the work is being conducted, the researcher is required to have sufficient interpersonal skills to create and nurture partnerships that share power and responsibility (29, 39, 50), at the same time ensuring patients’ right to participate are balanced with patients’ other life interests (29). Researchers require the skills to build on community strengths, and to release control of the study findings to the community or population studied (15).

***Communication Skills*:** Researchers can communicate difficult and complex concepts and ideas in understandable language (15, 29, 46, 51, 52); have interpersonal (53), mentorship (23), and listening skills (43); demonstrate transparency (23); are able to give feedback to volunteers (54), ensuring differences of opinion are expressed in a productive manner (16); support preparation of research reports and other documents (52); and, communicate with interested parties about the research (e.g., health organizations, decision- or policy-makers) (55).

***Teamwork – Group Process Skills****:* Researchers are able to build trusting relationships (28, 50, 53, 55, 56); to facilitate, mediate and encourage others in a group meeting (56); to create safe/supportive team atmospheres (33); clarify roles for each member of the research team (21, 28, 38, 46, 52); manage expectations (57); provide feedback to team members (21); manage differences by handling intense emotions (57); and, express differences in a productive manner (16). In some cases, researchers also have group process skills to be able to conduct focus groups (22).

***Project Management Skills:*** Researchers are able to create budgets for the costs of patient participation (38); prepare written contractual and legal agreements involving the conduct of research for team members (58); to prepare job descriptions (48); and, to present research results in a manner that is readily understood and meaningful (35).

***Conflict – Tension Management:*** Researchers have skills to identify areas of potential tension and resistance within the team (38); prevent conflicts or misunderstandings through facilitation/mediation and conflict resolution skills (15, 59); and, overcome tensions between differing research cultures (23).

***POR or POR-related skills:*** Researchers ensure their own competence in conducing POR (39) and assess their own personal learning needs (45). Additionally, researchers have skills to communicate openly with the research team (20); explain how public reviewers are used in review processes and how peer-review process works; and, to lead peer-review briefing meetings (60).

##### Attitude competencies

Patient-involved researchers’ attitudes represent an inclination to participate and collaborate, such as to build partnerships and relationships (21); establish consensus (50); share control (23, 50) while holding respect for differing perspectives (38); are sensitive to others’ concerns (42); value mutual trust (30) and establish relationships that exhibit openness and trust (55); commit to contributing to society, working toward the ‘greater good’ (22); respecting community values (15); and, the lived experiences of patients (39). Researchers support the use of creative methodologies (30) and have flexibility in conducting work (55, 61). Researchers hold a commitment to patient/public involvement (20); value research questions that are not researcher-driven (14); are willing to be accountable to the community (30) and to patients (29); respecting the ‘cultural gap’ between old and new ways of working (54); and, are inclined toward self-reflection and professional growth (24, 62).

Personal attributes: Researchers are described as being friendly, accommodating, patient, supportive, encouraging (63), approachable (20), optimistic (20), courteous (42), open (24), humble, curious (23), emotionally intelligent (20), and caring (42).

#### Patient stakeholder group

Seven themes were identified for knowledge competencies and eight themes for skills for patients.

##### Knowledge competencies

***Research Methods and Principles:*** Patients understand health research processes and methods (12–14, 18, 21, 34, 47, 56, 64–68); define and explain research approaches (12, 13, 18, 20, 21, 42, 47, 54, 62, 64–67, 69, 70); know how to be involved in the research process (71); understand participatory and community research methods (71); understand interviewing methods (66); define and use health research vocabulary (30, 42, 51, 54, 63, 65, 67, 70); understand instrumentation (13); comprehend the formation of research questions (14); understand commonly used quantitative methods (54, 62) and logic models (16); understand the process of literature reviews (68), literature analysis (65, 68), and journalistic writing (72); understand the meaning of data (62); understand the importance of research ethics (65); and, have a good understanding of the research project being conducted (73).

***Participatory Approaches****:* Patients understand their role within a research team and the roles of others (54, 63); can describe personal contributions to research projects (13, 42, 44, 47, 65, 67, 71, 72, 74); know how to bring personal perspective (42, 65, 67) and explain the needs of patients as respondents to research questions (55); understand the viewpoints and needs of other research partners (15, 63) and how to provide access to the patient population (13, 72).

***Cultural Competence and Context:*** Patients understand the basics of cultural competence (21, 56) and identify community needs, concerns, opinions and perspectives (58), as well as community resources (23). Patients have sufficient knowledge of the context to understand the place of the research project within the health and social care service systems (61, 75) and can identify current research activities within the context of work at national or local levels (18).

***Logistics:*** Patients have a realistic sense of the time required to participate in research when making a commitment to do so (12), and know the available supports and training opportunities (47).

***Understanding Evidence and Results:*** Patients understand evidence, its use and impact, and what can (or cannot) be achieved through research (47, 57, 62); understand the meaning of data (62), the full impact of study interventions (76), and can assess quality in research effort and outcomes (38).

***Knowledge About Phenomenon Under Study****:* Patients understand and define the phenomenon under study (49, 55, 69, 74); describe their own experience of the study phenomenon (58); and understand the needs of patients (55).

***Priority/Agenda Setting:*** Patients understand how research funding priorities are set (14).

##### Skills competencies

***Participatory skills:*** Patients are able to create partnerships that share power and responsibility equally and fairly (72); to collaborate in order to participate in all aspects of the study design; to establish rapport with study participants, and in so doing, identify community resources (52, 71).

***Communication skills:*** Patients are able to express personal experiences with the condition being studied in a compelling manner (46, 77); to ask questions and probes; to deliberate and argue about their own experiences and opinions (55); to listen to others with differing viewpoints (78) and to write (61) and communicate using technology (67).

***Research skills****:* Patients are able to read and comprehend research reports (12, 52, 65); develop the research design along with other members of the team (55); write research goals (27, 52, 62); reflect the experiences of the study population in the research questions (52); identify research gaps in knowledge from a consumer perspective (76); recruit research participants (33); and, place patient stories in the research process within the context of their own life experience (33). In some cases, patients need skills to write consent forms, questionnaires, or interview schedules (49) and use computers and software programs (18). Depending on the research team and approach, patients may have skills to review research protocols (79), collect data (49, 80), interview participants (80), present data (72), collaborate in data analysis (33, 80), identify patient-important themes in data (49), participate in ethical debates (58), and work with others to establish research networks (72).

***Teamwork/Group Process****:* Patients can define their own role in the research project (29, 46) and raise issues important to patients (29); work effectively in a group to keep a patient-centred approach at the forefront of the team’s awareness (63) and provide feedback to the team (29); build relationships (56) or teams (80); mentor others (23); mediate in the group setting (23); and handle intense emotions with those who have differing views (57).

***Project Management****:* Patients can manage projects or aspects of the work (for those who take on managerial roles) (80).

***Conflict/tension Management****:* Patients are able to deal productively with conflicts that arise (18).

***Priority Setting****:* Patients can influence what is being investigated currently and in the future (55).

***Evidence and Results****:* Patients can interpret and evaluate research findings (52, 55) and critically assess risks and benefits of treatments (70). In relation to research findings, patients can support the dissemination of research results (72, 79, 81) and collaborate with other team members to create a shared set of reliable sources of evidence (76).

##### Attitude competencies

Patients are characterized as individuals having interest in research outcomes (21), commitment to contributing to society (22), and willingness to commit to long-term projects (15, 30, 63). They have an interest in contributing to healthcare improvement (76). They are noted to value mutual benefits in collaborations (15), respect the complexities of a research project (35); are able to represent more than their personal individual experiences (30); commit to shared decision-making (37); respect differing skills (38); and, value the knowledge of researchers (30).

Personal attributes: Patients have personal attributes of confidence (82), emotional intelligence (20), good communication (63), humility, curiosity (23), and hold a constructive and positive attitude (46). One source expressed the view than an essential attribute is that the patient have no professional healthcare or research background (45) and another (72) stated “the more professional the patient research partners become, the greater chance they will lose touch with their fellow patients” (p.408).

#### Healthcare provider and health system decision-maker stakeholder groups

In the literature, there are identical patient-involved research competencies for providers and decision-makers. Two themes were identified for knowledge and two for skills competencies.

##### Knowledge competencies

***Research Methods and Principles****:* These stakeholders understand patient involvement in research and are able to list research goals and identify the needs of research partners (15); understand the research process, the purpose of health research, the funding process, the roles and responsibilities of those conducting patient-involved research (45); and, have knowledge of POR principles and understand the benefits of POR or POR-related work (44, 45).

***Participatory Approach****:* These stakeholders understand the need to collaborate with those who have differing perspectives while handling intense emotions that arise (57). In addition, all involved have knowledge and understanding of team development, decision-making, and communication methods (45).

##### Skills competencies

***Critical Thinking:*** These stakeholders can apply a critical appraisal lens to research projects (76).

***Teamwork/Group Process****:* These stakeholders collaborate with others, especially when dealing with differing perspectives and situations where strong emotions are expressed (57); and, listen and look beyond one’s own world view (45).

##### Attitude competencies

Providers and decision-makers value research outcomes (21); want to contribute to society (22); hold a commitment to openness, respect, trust, and engagement of all stakeholders (55); respect differing skills (38), community values (15), and commitment to long-term projects (63); value trust (30), effective communication (51), relationships that are open and respectful (55), and the mutual benefits achieved through such relationships (15).

Personal attributes: These stakeholders are described as having emotional intelligence (20); being culturally and politically aware (20); having an attitude of curiosity and humility (23); and, having a constructive and proactive nature (46).

## Discussion

The competencies provide both a comprehensive and inclusive view of the thinking of many individuals engaged in patient-involved research. We note that some of the competencies apply to a particular research method, so it is challenging to determine what competencies are ‘essential’ and what competencies are desirable for each research team. The competencies we found in the literature need to be considered with discretion. We also note that some of the competencies reported were noted only once.

Our reflections on the competencies have been enriched by feedback from an expert Group of Advisors, as it is apparent that the relevance or appropriateness of any of the competencies may be disputed. We comment below on the competencies for each of the stakeholder groups.

### Researcher competencies

In addition to research competencies, POR and POR-related or patient-involved researchers need to know how to engage patients, manage project logistics, and collaborate with others in setting research directions. Thus, the researcher uses communication, interpersonal, managerial, and fiscal skills in addition to those in research. These findings are supported in a recently published report of a group of 18 experienced researchers who reflected on the ‘lessons learned’ from conducting partnered research (83). These researchers concluded that good communication, treating all with respect and inclusivity, and ensuring that there is funding for all who participate are essential for project success.

For researchers, however, the need for group work, attention to group process, collaboration, and sharing of power, create uncertainties particularly regarding accountability to research, science, funding bodies and timelines available to conduct studies. For example, feedback from our Advisors included recognition that it is not possible for every team decision to be made through consensus. Yet, this feedback also documented a reluctance among some that the researcher be the individual with decision-making power. Thus, the challenge of being the ‘leader’ with accountability comes up against a process that expects - or even mandates - group decisions, power sharing and equality among all team members. Our sense is that researchers entering into this work need to be able to work within these uncertainties and have commitment to the ideals of patient participation with the belief that such work creates a public good.

### Patient competencies

There was a strong convergence between the thoughts of our own team members and the comments from our Advisors that the identified patient competencies present too many requirements for knowledge and skills, making the statement of competencies either elitist or unattainable. One of our Advisors commented that we should not be trying to make our patient partners into researchers themselves. We conclude that not every identified competency, even if important for an individual study, is needed by every patient in every study. The challenge for research teams is to take these reported competencies and draw out what is needed for their particular study. The most important contributions a patient brings is having lived experience of the condition being studied, experience of navigating the health system, and the willingness to share those experiences. Additional competencies would include an understanding of research and the research methods being used and a desire to contribute to the research team effort. However, we have several considerations in terms of patient competencies.

1. Training Programs: Training programs need not be devised for *all* patients for *all* research programs. Current training programs contain many of the knowledge competencies found in our review, yet these programs may be trying to teach too much content. Existing training programs focus on knowledge, not the skills, attitudes or personal characteristics supportive to work within a research team. The question then arises of how a research team determines what competencies patients actually need in order to participate. Feedback from our Advisors suggested that many competencies are best learned on-the-job by having an individual engage in research work, not in training programs. A research team would then need to decide how the issue of training will be managed in context of that team’s work. We suggest patients themselves should determine their own learning needs in the context of their project team.
2. Relationship and communication: There are several, seemingly reasonable, competencies for patients that deal with the ability to collaborate, work within a group, or find consensus; yet, there is concern about these ideas. Our Advisors questioned how a team would address competencies such as ‘having empathy’ when it may not be possible to teach ‘empathy’ as a skill. One solution might be that some competencies related to ‘empathy’ or ‘willingness to find consensus’ be used for individuals to assess their own readiness to participate in a POR or POR-related research team, rather than attempt to incorporate them into a training program.
3. Patient engagement, patient-involved research, and the patient’s role: Jennifer Johannesen, an author, educator, patient advocate, and critic who herself was a parent of a child with disabilities, consults with governments and institutions on matters related to patient-involved research. She raises concerns about how patients are used in teams. She critiques the patient/public engagement movement as a means to co-opt patients as participants in research carried out according to organizational polices and plans. (https://johannesen.ca/2018/09/the-trouble-with-patient-and-public-involvement-ppi-keynote-at-cochrane-colloquium-2018/). She notes the patient engagement enterprise (as she refers to it) is very different from patient grassroots movements to direct changes in healthcare. She expresses concerns that much of the selection and teaching of patients and public members is to ensure individuals in these roles are compliant and willing to sustain a large research industry. Her reasoning cautions us that the stated need for relationship skills could be used as a method of eliminating dissent as much as it is a way of bringing the right people into a project. The distinction between eliminating dissent and providing useful service to research may be the difference between a noble or an inappropriate use of citizen input. If so, it is incumbent on the research team (and maybe the funder) to evaluate the ways in which patients are being engaged. The idea of finding an appropriate ‘critical friend’, one familiar with the issues yet supportive of the overall approach, may be a way for research teams to address these important concerns while moving forward with the work.
4. Patient Research Skills: We are hesitant to accept the inclination in the literature that patients on a research team need to have an exhaustive set of research competencies or to learn the research process in order to participate in a research team. For example, we do not expect all patients to be able to interpret and evaluate research, analyze data, or critically assess risks and benefits of treatments. Instead, our expectations from patients highlight their ability to understand the purpose of the study, to share their relevant experiences with the research team members, and have the willingness to contribute to the dissemination of the study findings. However, we also note that there are circumstances which raise different questions such as a situation where the patients or community partners become researchers themselves. When the patient or community partners become the researcher, are they taking on the ‘researcher role’ and thereby needing to meet the researcher competencies? Can, then, a patient be both a researcher and a patient partner in the study? The literature does not address these questions and we recommend that each research team consider the best way forward for their own project.
5. Patient Professional Experience: The last issue regarding patient competencies is a debate over the stated characteristics of the patient team member as one who has no healthcare, health service, or research background (as described by Abma & Broerse (55) in our review) and incorporated into the language of CIHR (45). Some of our team members and our Advisors are not willing to accept that an individual managing a chronic and severe health condition would be unable to accurately express a patient perspective simply because that person has had professional experience. This debate is not resolved, but one that research team members need to consider when deciding which individuals meet the needs of a particular research project.

### Provider and decision-maker competencies

Competencies of these groups have certainly not attracted much attention. Beyond understanding a patient-involved approach to research and knowing the roles and activities of research team members, the most pertinent competencies seem to be holding positive attitudes toward research and having interest in research outcomes that have potential of making a difference to society. We and our Advisors noted that decision-makers have the ability to create conditions in institutions that encourage or discourage partnered research. This notion is consistent with a recent report of patient engagement in quality evaluations where it was noted that ‘top-down’ approaches to support partnered work can change institutional culture, as decision-makers can not only provide funding, they can align partnered activities with strategic goals (84). Similarly, healthcare providers can support patient-involved research by identifying gaps in knowledge, using patient knowledge to assist in recruitment of patient partners, and becoming involved in translating research into practice. Based on our review, the actual competencies for these two groups remain largely unknown.

### Limitations and strengths

As a scoping review, our work has limitations. There was no effort made to assess the quality of research, evidence or science behind the competencies. Our findings provide an inclusive statement of competencies for stakeholders, yet, does not prioritize one competency over another; research on what makes a team effective would be required to do so. A systematic literature review would seem an obvious next step, however our review indicates there is a limited number of research studies on these competencies, and those that exist are qualitative studies seeking to understand and/or describe the processes of patient-involved research. Furthermore, searching the literature is challenging as there are no common keywords/word phrases, or dedicated journals. The lack of a common vocabulary is problematic, as we have found and is also noted by researchers completing a systematic review of patients participating as co-researchers in health research (85).

The strengths of our review include the rigorous process of a knowledge synthesis following standard steps and stages, the involvement of an interdisciplinary research team, and obtaining feedback from a Group of Advisors comprised of all stakeholder groups.

### Recommendations

We recommend that these competencies be used by any research team to evaluate its abilities to conduct patient-involved research. Likewise, the competences can be used by individuals as a self-assessment of their readiness to participate in a research team. Thus, learning needs can be identified and addressed.

We found that there is a clear need to address the lack of a common vocabulary for POR and POR-related or patient-involved research work. Authors of a systematic review of patients (85) as co-researchers commented: “Identifying appropriate search terms was a challenge, as hardly any available standard keywords incorporated the phenomena we wanted to explore. Several unproductive test searches provided enormous numbers of hits but no relevant publications.” (p.3) a finding that exactly replicates our own experience. A concept analysis of terms currently used would be a helpful step in identifying the core concepts. Further, we note that even the basic terms used by authors in the field may lack clarity. Is ‘patient-involved research’ the same as ‘patient-engaged research’? Are the terms defined differently in different geographical areas? Is ‘patient-oriented research’ in Canada the same as ‘patient-engaged research’ in the UK or ‘patient-involved research’ in Australia? What does ‘community-based’ research mean? And in what settings? Unless and until there emerges a global consensus on use of terms, this lack of clarity will inhibit full development of the science of patient-involved research and impact the sharing of data and experiences across international boundaries. We recommend that those working in this area consider the GRIPP2 checklist (51) for reporting to build a common understanding of this field of research.

## Conclusions

The findings of our scoping review are indicative of a research area that has not yet been fully documented or studied. There remain many unanswered questions related to each of the stakeholder competencies needed by a POR team. We encourage research on issues raised in our reflections, such as the ambiguities associated with the decision-making of a team unable to achieve consensus, the sense that an experienced professional who is a patient can or cannot present the patient voice, and the emerging roles for healthcare providers and decision-makers in this field. Additionally, the health outcomes and benefits of patient-involved research have not been fully explored. To address the issue of common vocabulary, we recommend publications should strive to identify and use keywords that would assist in this area of research and scholarship.

## Data Availability

The datasets generated and analyzed during this current review paper are available in the University of Victoria (Victoria, British Columbia, Canada) repository and can be located at: https://dspace.library.uvic.ca//handle/1828/10917.

## Abbreviations

CIHR: Canadian Institutes of Health Research
CINAHL: The Cumulative Index of Nursing and Allied Health Literature, a bibliographic database
Embase: Bibliographic database for biomedical research
GRIPP2: Guidance for Reporting Patient and Public Involvement, short form; a checklist used for reporting patient and public involvement in health and social care
IAP_2_: International Association for Public Participation
INVOLVE: A national advisory group to support patient involvement in the NHS
MEDLINE: Bibliographic database for life sciences and biomedical information
NHS: The National Health Service of the UK
Patient-involved research: Health research conducted in a manner to include patients, family members and/or community partners as members of a research team along with researchers and other health professionals
POR: Patient Oriented Research, a Canadian initiative to support patient engagement in health research
POR-related research: Health research in countries outside of Canada that strives for patient engagement in the research process
Web of Science: Website that provides access to multiple bibliographic databases

## References

[1] Canadian Institutes of Health Research (CIHR) (2015). Strategy for Patient-Oriented Research Putting Patients First: Capacity development framework (Appendix 2 - Core Competencies):Canadian Institutes of Health Research (CIHR).

[2] Goudreau J, Pepin J, Dubois S, Boyer L, Larue C, Legault A (2009). A second generation of the competency-based approach to nursing education. Int J Nurs Educ Scholarsh.

[3] Arksey H, O'Malley L (2005). Scoping studies: towards a methodological framework. Int J Soc Res Methodol.

[4] Levac D, Colquhoun H, O'Brien K (2010). Scoping studies: advancing the methodology. Implement Sci.

[5] Colquhoun HL, Levac D, O'Brien KK, Straus S, Tricco AC, Perrier L (2014). Scoping reviews: time for clarity in definition, methods, and reporting. J Clin Epidemiol.

[6] Mallidou AA, Frisch N, Doyle-Waters MM, MacLeod MLP, Ward J, Atherton P (2018). Patient-oriented research competencies in health (PORCH) for patients, healthcare providers, decision-makers and researchers: protocol of a scoping review. Syst Rev.

[7] Rogers M, Bethel A, Boddy K (2017). Development and testing of a medline search filter for identifying patient and public involvement in health research. Health Inf Libr J.

[8] Whittemore R, Knafl K (2005). The integrative review: updated methodology. J Adv Nurs.

[9] Cooper H (1998). Synthesizing research: a guide for literature reviews.

[10] Mallidou AA (2014). Mapping the landscape of knowledge synthesis. Nurs Manag (Harrow, London, England).

[11] Hsieh H-F, Shannon SE (2005). Three approaches to qualitative content analysis. Qual Health Res.

[12] Bayliss K, Starling B, Raza K, Johansson EC, Zabalan C, Moore S (2016). Patient involvement in a qualitative meta-synthesis: lessons learnt. Res Involv Engagem.

[13] Duffett L (2017). Patient engagement: what partnering with patient in research is all about. Thromb Res.

[14] Fleurence R, Selby JV, Odom-Walker K, Hunt G, Meltzer D, Slutsky JR (2013). How the patient-centered outcomes research institute is engaging patients and others in shaping its research agenda. Health Affairs (Project Hope).

[15] Ahmed SM, Palermo AGS (2010). Community engagement in research: frameworks for education and peer review. Am J Public Health.

[16] May M, Law J (2008). CBPR as community health intervention: institutionalizing CBPR within community based organizations. Prog Community Health Partnersh.

[17] Columbia University: Mailman School of Public Health (2017). Patient Oriented Research.

[18] INVOLVE (2017). Training for research panel members.

[19] International Collaboration on Participatory Health Research (ICPHR) (2017). Promoting the science and enhancing the impact of participatory health research.

[20] INVOLVE (2016). NIHR public involvement leads’ national meeting.

[21] Shippee ND, Domecq Garces JP, Prutsky Lopez GJ, Wang Z, Elraiyah TA, Nabhan M (2015). Patient and service user engagement in research: a systematic review and synthesized framework. Health Expect.

[22] Dudley L, Gamble C, Allam A, Bell P, Buck D, Goodare H (2015). A little more conversation please? Qualitative study of researchers’ and patients’ accounts of training for patient and public involvement in clinical trials. Trials..

[23] Horowitz CR, Robinson M, Seifer S (2009). Community-based participatory research from the margin to the mainstream: are researchers prepared?. Circulation..

[24] Braye S, Preston-Shoot M (2005). Emerging from out of the shadows? Service user and carer involvement in systematic reviews. Evid Policy: J Res Debate Pract.

[25] INVOLVE (2014). Public involvement in research applications to the national research ethics service: comparative analysis of 2010 and 2012 data.

[26] Healthtalk.org-f. Researchers’ experiences of patient & public involvement: Researchers’ examples of the value and impact of involvement. Oxford: Oxford University. Available from: http://www.healthtalk.org/peoples-experiences/medical-research/researchers-experiences-patient-public-involvement/researchers-examples-value-and-impact-involvement.

[27] INVOLVE. How to get actively involved in NHS, public health and social care research. So, what is it all about? National Health Service, National Institute for Health Research; 2007b. Available from: http://www.invo.org.uk/wp-content/uploads/2011/12/PIP1whatisitallabout.pdf.

[28] Shaywitz DA, Martin JB, Ausiello DA (2000). Patient-oriented research: principles and new approaches to training. Am J Med.

[29] Bailey S, Boddy K, Briscoe S, McHugh C, Stone T, East A (2013). Involving disabled children and young people as partners in research: a systematic review 2013; Developmental medicine and child neurology. Conference: 25th annual meeting of the European academy of childhood disability.

[30] McCormick S, Brody J, Brown P, Polk R (2004). Public involvement in breast cancer research: an analysis and model for future research. Int J Health Serv.

[31] Healthtalk.org-d. Researchers’ experiences of patient & public involvement: Learning from experience of involving patients and the public. Oxford: Oxford University. Available from: http://www.healthtalk.org/peoples-experiences/medical-research/patient-public-involvement-researchers/learning-experience-involving-patients-and-public.

[32] INVOLVE (2012). Developing training and support for public involvement in research.

[33] Nierse CJ, Schipper K, van Zadelhoff E, van de Griendt J, Abma TA (2012). Collaboration and co-ownership in research: dynamics and dialogues between patient research partners and professional researchers in a research team. Health Expect.

[34] Healthtalk.org-c. Researchers’ experiences of patient & public involvement: Finding people to involve in research Oxford: Oxford University; [Available from: http://www.healthtalk.org/peoples-experiences/medical-research/researchers-experiences-patient-public-involvement/finding-people-involve-research.].

[35] Consumers Health Forum of Australia (CHFA) (2017). Consumers Health Forum of Australia.

[36] Health Research Authority I (2017). Impact of public involvement on ethical aspects of research.

[37] Kelly PJ (2005). Practical suggestions for community interventions using participatory action research. Public Health Nurs.

[38] Morrow E, Ross F, Grocott P, Bennett J (2010). A model and measure for quality service user involvement in health research. Int J Consum Stud.

[39] Telford R, Boote JD, Cooper CL (2004). What does it mean to involve consumers successfully in NHS research? A consensus study. Health Expect.

[40] Alberta SPOR Support Unit (2017). Alberta engagement platform: resources to support patient engagement: Alberta innovates.

[41] Alberta SPOR Support Unit (2017). POR training: session 4: innovative methods for patient-oriented research: part 1: Eventbrite.

[42] Healthtalk.org-b. Researchers’ experiences of patient & public involvement: Colleague's attitudes to patient and public involvement Oxford, UK: Oxford University; [Available from: http://www.healthtalk.org/peoples-experiences/medical-research/patient-public-involvement-researchers/colleagues-attitudes-patient-and-public-involvement.].

[43] Healthtalk.org-g. Researchers’ experiences of patient & public involvement: Skills needed for involvement Oxford, UK: Oxford University; [Available from: http://www.healthtalk.org/peoples-experiences/medical-research/patient-public-involvement-researchers/skills-needed-involvement.].

[44] Alberta SPOR Support Unit (2017). Alberta Innovates.

[45] Canadian Institutes of Health Research (CIHR) (2016). Foundations for Patient-Oriented Research, National Curriculum for POR.

[46] de Wit M, Berlo SE, Aanerud GJ, Aletaha D, Bijlsma JW, Croucher L (2011). European league against rheumatism recommendations for the inclusion of patient representatives in scientific projects. Ann Rheum Dis.

[47] INVOLVE (2017). Training for advisory group members.

[48] Maybee A, Clark B, McKinnon A, Angl EN (2016). Patients Canada: citizens partnering in health research: researcher orientation to patient partnership.

[49] Brett J, Staniszewska S, Mockford C, Seers K, Herron-Marx S, Bayliss H (2010). The PIRICOM study: a systematic review of the conceptualisation, measurement, impact and outcomes of patients and public involvement in health and social care research.

[50] Hubbard G, Kidd L, Donaghy E, McDonald C, Kearney N (2007). A review of literature about involving people affected by cancer in research, policy and planning and practice. Patient Educ Couns.

[51] Staniszewska S, Jones N, Newburn M, Marshall S (2007). User involvement in the development of a research bid: barriers, enablers and impacts. Health Expect.

[52] Reed J, Weiner R, Cook G (2004). Partnership research with older people - moving towards making the rhetoric a reality. J Clin Nurs.

[53] Backhouse T, Kenkmann A, Lane K, Penhale B, Poland F, Killett A (2016). Older care-home residents as collaborators or advisors in research: a systematic review. Age Ageing.

[54] Howe A, MacDonald H, Barrett B, Little B (2006). Ensuring public and patient participation in research: a case study in infrastructure development in one UK Research and Development consortium. Prim Health Care Res Dev.

[55] Abma TA, Broerse JEW (2010). Patient participation as dialogue: setting research agenda. Health Expect.

[56] Katigbak C, Foley M, Robert L, Hutchinson MK (2016). Experiences and lessons learned in using community-based participatory research to recruit Asian American immigrant research participants. J Nurs Scholarsh.

[57] Coon JT, Gwernan-Jones R, Moore D, Richardson M, Shotton C, Pritchard W (2016). End-user involvement in a systematic review of quantitative and qualitative research of non-pharmacological interventions for attention deficit hyperactivity disorder delivered in school settings: reflections on the impacts and challenges. Health Expect.

[58] European Patients’ Academy (EUPATI) (2017). European Patients Academy on therapeutic innovation.

[59] Hubbard G, Kidd L, Donaghy E (2008). Involving people affected by cancer in research: a review of literature. European Journal of Cancer Care.

[60] INVOLVE (2017). Training for public reviewers.

[61] Healthtalk.org-e. Researchers’ experiences of patient & public involvement: Practical advice for involvement. Oxford: Oxford University. Available from: http://www.healthtalk.org/peoples-experiences/medical-research/patient-public-involvement-researchers/practical-advice-involvement.

[62] Meyer MC, Torres S, Cermeno N, MacLean L, Monzon R (2003). Immigrant women implementing participatory research in health promotion. West J Nurs Res.

[63] Robbins M, Tufte J, Hsu C (2016). Learning to “swim” with the experts: experiences of two patient co-investigators for a project funded by the Patient-Centered Outcomes Research Institute. Permanente J.

[64] Arkind J, Likumahuwa-Ackman S, Warren N, Dickerson K, Robbins L, Norman K (2015). Lessons learned from developing a patient engagement panel: an OCHIN report. J Am Board Fam Med.

[65] Healthtalk.org-a. Patient and public involvement in research: Training and learning Oxford: Oxford University; [Available from: http://www.healthtalk.org/peoples-experiences/improving-health-care/patient-and-public-involvement-research/training-learning-and-support.].

[66] INVOLVE (2017). Training for peer interviewers.

[67] INVOLVE (2017). Training for steering group members.

[68] Cardiff University (2017). Learning online: searching and researching.

[69] Kirwan JR, de Wit M, Frank L, Haywood KL, Salek S, Brace-McDonnell S (2017). Emerging guidelines for patient engagement in research. Value Health.

[70] Cardiff University (2017). Making sense of health evidence: the informed consumer.

[71] INVOLVE (2007). How to get actively involved in NHS, public health and social care research: getting started UK: National Health Service, National Institute for Health Research.

[72] Abma TA, Nierse CJ, Widdershoven GAM (2009). Patients as partners in responsive research: methodological notions for collaborations in mixed research teams. Qual Health Res.

[73] Maybee A, Clark B, McKinnon A, Angl EN (2016). Patients Canada: patients as partners in research: planning guidelines.

[74] INVOLVE (2012). Training case study nine: informal approach to assessing training needs.

[75] National Health Service UK (2017). Patient research ambassador initiative: putting people at the heart of research.

[76] Cochrane Consumer Network (2017). Trusted evidence. Informed decisions. Better Health.

[77] Ismail S (2009). Participatory Health Research: international observatory on Health Research systems. Technical report.

[78] James Lind Alliance (2017). James Lind Alliance.

[79] Chalmers JD, Timothy A, Polverino E, Almagro M, Ruddy T, Powell P (2017). Patient participation in ERS guidelines and research projects: the EMBARC experience. Breathe..

[80] Cornes M, Peardon J, Manthorpe J, Yo PT (2008). Wise owls and professors: the role of older researchers in the review of the National Service Framework for older people. Health Expect.

[81] Blair T, Minkler M (2009). Participatory action research with older adults: key principles in practice. Gerontologist..

[82] Alberta SPOR Support Unit: Summer Institute (2017). Working together: A spotlight on Patient-Oriented Research: Alberta Innovates, Summer Institute.

[83] Witteman HO, Chipenda Dansokho S, Colquhoun H, Fagerlin A, Giguere AMC, Glouberman S (2018). Twelve lessons learned for effective research partnerships between patients, caregivers, clinicians, academic researchers and other stakeholders. J Gen Intern Med.

[84] Bombard Y, Baker GR, Orlando E, Fancott C, Bhatia P, Casalino S (2018). Engaging patients to improve quality of care: a systematic review. Implement Sci.

[85] Malterud K, Elvbakken K. Patients participating as co-researchers in health research: A systematic review of outcomes and experiences. Scand J Public Health, OnlineFirst, 10.1177/1403494819863514. 10.1177/1403494819863514 31319762

